# Chediak-Higashi Syndrome in Accelerated Phase Masquerading as Acute Leukemia

**DOI:** 10.4274/tjh.2015.0446

**Published:** 2016-12-01

**Authors:** Mili Jain, Ashutosh Kumar, Uma Shankar Singh, Rashmi Kushwaha

**Affiliations:** 1 King George’s Medical University, Department of Pathology, Uttar Pradesh, India

**Keywords:** Chediak Higashi syndrome, Giant granules, Immunodeficiency

We present a 3-year-old female born of a consanguineous marriage with the complaints of high-grade fever, petechial spots, abdominal distension, and lymphadenopathy for 20 days. She had pallor, hypopigmented hairs, petechial rashes, and palpable lymph nodes (up to 1 cm) in the bilateral inguinal and cervical region. Systemic examination revealed hepatosplenomegaly. Her hematological profile was as follows: hemoglobin of 8.4 g/dL, normocytic normochromic red cell indices, platelet count of 11x10^9^/L, total leukocyte count of 7x109/L with increased lymphocytes (68.5%), and lactate dehydrogenase raised at 796 IU/L. The peripheral blood smear examination revealed giant granules in neutrophils, lymphocytes, and monocytes (Figure 1). Bone marrow examination revealed similar granules in myeloid precursors with moderate hemophagocytosis. Examination of the hair shafts showed large melanin granules (Figure 2). Her liver function tests, kidney function tests, and chest X-ray results were within reference ranges. She was diagnosed with Chediak-Higashi syndrome (CHS) in the accelerated phase.

CHS is a rare autosomal recessive disorder (gene CHS1/LYST) [1]. The clinical picture includes partial oculocutaneous albinism, abnormal bleeding time, peripheral neuropathy, and recurrent severe bacterial infection [2]. The giant lysosomal granules (formed as a result of cytoplasmic injury, phagocytosis, and fusion due to microtubular defects) in white blood cells are pathognomonic for diagnosis [3].

## Figures and Tables

**Figure 1:**
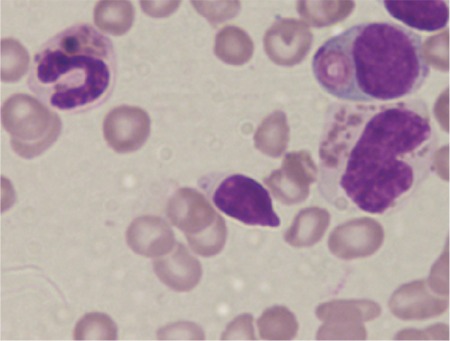
Peripheral blood smear with Leishman stain at 400x: giant granules in neutrophils and lymphocytes.

**Figure 2:**
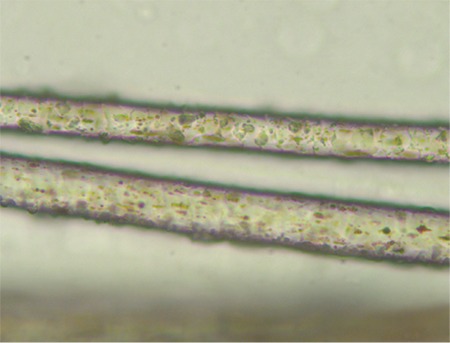
Hair follicles at 400x with irregularly sized melanosomes.
